# Treatment Pathways and Outcomes in Patients with BMI ≥ 50 kg/m^2^: Conservative Treatment, Immediate Surgery or Stepwise Surgical Approach

**DOI:** 10.1007/s11695-025-08102-1

**Published:** 2025-07-18

**Authors:** Sara Notz, Rainer Grotelueschen, Julia Pape, Bjoern-Ole Stueben, Louisa Stern, Julia Gerullies, Jonas Wagner, Anne Lautenbach, Jakob Robert Izbicki, Thilo Hackert, Philipp Busch, Anna Dupree, Dieter Weber, Oliver Mann, Gabriel Plitzko

**Affiliations:** 1 Department of General, Visceral and Thoracic Surgery, University Medical Center Hamburg-Eppendorf, Hamburg, Germany; 2 Department of General Surgery, Royal Perth Hospital, Perth, Australia; 3 Department of General-, Visceral- and Transplant Surgery, Essen University Hospital, Essen, Germany; 4 Endocrinology, Diabetes, Obesity, and Lipid Disorders, Outpatient Center of the University Medical Center Hamburg Eppendorf, Hamburg, Germany

**Keywords:** Metabolic and bariatric surgery, Conservative treatment, Stepwise approach, Weight loss outcomes, Comorbidity resolution, Obesity treatment pathways

## Abstract

**Background:**

Bariatric surgery is the most effective treatment for patients with BMI ≥ 50 kg/m^2^, yet preoperative conservative therapy is often mandated. The benefit remains controversial. This study compared outcomes of conservative treatment, immediate surgery, and surgery after conservative therapy in patients with BMI ≥ 50 kg/m^2^.

**Methods:**

All patients with BMI ≥ 50 kg/m^2^ at a German bariatric center (2015–2021) were retrospectively categorized into: Non-Surg (conservative therapy only), Surg-First (immediate surgery), and Step-Treat (initial conservative therapy followed by surgery). Primary outcomes at 6, 12, and 24 months included weight loss, resolution of comorbidities (hypertension, type 2 diabetes, dyslipidemia, obstructive sleep apnea), Quality of Life and overall outcome (SF-BARI QOL score).

**Results:**

Of 918 screened patients, 538 met inclusion criteria: Non-Surg (*n* = 203), Surg-First (*n* = 225), Step-Treat (*n* = 110). After 6 months, median % total weight loss (%TWL) and % excess weight loss (%EWL) were significantly higher in Surg-First (%TWL: 23.6% vs. 0%; %EWL: 42% vs. 0%; both *P* < .001) vs. Non-Surg. Surg-First showed higher diabetes remission (54.1% vs. 21.2%; *P* < .001) and fewer de novo diabetes cases. Compared to Surg-First, Step-Treat showed similar short-term weight loss but lower %TWL and %EWL at 12 and 24 months. Severe postoperative complications (Grade IIIb) were more common in Step-Treat (11.8% vs. 3.6%; *P* < .001), and SF-BARI QOL scores were higher in Surg-First.

**Conclusion:**

In patients with BMI ≥ 50 kg/m^2^, immediate bariatric surgery resulted in superior weight loss, improved comorbidity resolution, and fewer complications compared to conservative therapy alone or a stepwise approach, supporting direct surgical treatment in this population.

## Introduction

In the developed world, obesity is one of the greatest public health problems. The global prevalence of obesity has increased substantially over the past 40 years, from 3 to 11% among men and from 6 to 15% among women from 1975–2016. Over the same time period, there has also been a marked increase in children from < 1% to 6–8% obesity prevalence [1]. In industrialized nations, obesity can now be considered a public health crisis. 67% of the US population is currently either overweight or obese, with the prevalence in most European countries ranging between 40 and 50% [2].

A systematic analysis on global health burdens showed high body mass index (BMI) to be among the top five risk factors in terms of attributable deaths and disability-adjusted life-years [3]. Obesity is associated with an increased risk for type 2 diabetes, hypertension, dyslipidaemia, cardiovascular diseases, certain types of cancer, and mortality [4].

Treatment options for obesity include non-surgical treatment and bariatric surgery. The non-surgical treatment usually entails multidisciplinary therapeutic approaches comprising behavioural therapy, dietary education and changes, increased physical activity, and pharmacotherapy [5]. Surgery has been shown to be the most effective treatment for obesity in terms of long-term weight loss, improvement of co-morbidities and quality of life [6–8]. Current IFSO (International Federation for the Surgery of Obesity and Metabolic Diseases) guidelines recommend considering surgery in patients aged 18–60 years with a BMI ≥ 35 kg/m^2^ regardless of co-morbidities or with a BMI 30–34.9 kg/m^2^ with metabolic disease [9].

Metabolic and bariatric surgery (MBS) include procedures, such as gastric banding, sleeve gastrectomy (SG), and Roux-en-Y gastric bypass (RYGB), and malabsorptive procedures such as distal Roux-en-Y bypass, bilio-pancreatic diversion with/without duodenal switch (BPD-DS), or one-anastomosis gastric bypass.

Multiple studies have compared outcomes in terms of weight loss after RYGB for patients with obesity class IV and V (BMI ≥ 50 kg/m^2^, BMI ≥ 60 kg/m^2^) versus patients with obesity class III (BMI ≥ 40 kg/m^2^), with some series showing similar outcomes [10, 11], whereas others describe inferior weight loss for patients with obesity class IV and V [12–16].

However, patients with BMI ≥ 50 kg/m^2^ present several unique challenges, with surgery being technically more difficult as BMI increases, frequently making RYGB impossible due to a shortening of the small bowel mesentery preventing an adequate mobilisation of the small intestine, with the only remaining option being a SG.

It would therefore appear beneficial to facilitate preoperative weight loss through conservative multidisciplinary therapy. However, the efficacy of preoperative conservative treatment is debatable. Two meta-analyses including 2869 patients and a retrospective comparative analysis of 2628 patients report a mean weight loss of only 1.7–5.3 kg after 6 months of treatment [3, 17, 18]. Additionally, data is lacking as to whether this would translate into improved postoperative outcomes. Several studies could not demonstrate an influence of a preoperative weight loss on the postoperative weight outcome [19–22].

Nevertheless, many health insurance providers and several governmental regulatory authorities mandate a preoperative multidisciplinary conservative therapy. However, some national guidelines, such as the German Guideline for the Surgery of Obesity and metabolic diseases and the British NICE guideline, state a BMI ≥ 50 kg/m^2^ as the criterion which qualifies patients for an direct surgical treatment [23, 24],

The aim of this study was to compare short- and mid-term treatment outcomes in patients with a BMI ≥ 50 kg/m^2^ who underwent either conservative treatment alone, immediate bariatric surgery, or initial conservative management followed by surgery.

## Methods

### Study Design and Data Collection

All patients with a BMI ≥ 50 kg/m^2^ who presented to Hamburg University Hospital between January 1, 2015, and December 31, 2021, were included in this study. Patients with incomplete follow-up, missing follow-up data and those who either received a revisional procedure after previous MBS or a GLP-1 receptor agonist therapy were excluded.

Patients were categorized into three groups based on their treatment approach. Those who exclusively underwent conservative, non-surgical therapy were assigned to the *Non-Surg* group, while those who proceeded directly to MBS without prior structured conservative therapy were classified as the *Surg-First* group. Patients who initially received conservative therapy but later underwent MBS were included in the *Step-Treat* group. The allocation to the *Non-Surg* or *Surg-First* group was determined by patient preference and the availability of insurance coverage for bariatric surgery. In the *Step-Treat* group, the decision to proceed with MBS was made when adequate weight loss could not be achieved through conservative therapy, and patients consented to surgical intervention as the preferred therapeutic option. In all patients who underwent MBS either SG or RYGB was performed.

Before surgery each patient was evaluated by a multidisciplinary team of endocrinologists, mental health professionals, dieticians, physical therapists and surgeons. According to the German Guidelines for the Surgical Treatment of Obesity, patients with a BMI > 40 kg/m^2^ alone or BMI > 35 kg/m^2^ and obesity-related comorbidities qualified for surgery. The choice of procedure was based on BMI, comorbidities, medication, intraoperative factors and patient´s preference (provided there were no contraindications).

Data on weight, obesity related co-morbidities (hypertension, type 2 diabetes [T2DM], dyslipidemia, obstructive sleep apnea syndrome [OSAS]) and medication were collected at the beginning and end of the conservative therapy, before the procedure, and at 6, 12, and 24 months follow-up. The duration of the operation, length of hospital stay (LOS), length of ICU stay, perioperative complications and quality of life (QOL) were also recorded. For weight loss outcomes, percent of total weight loss (%TWL) and percent excess weight loss (%EWL) were calculated [25].

In the first step, the *Non-Surg* group was compared to the *Surg-First* group in terms of weight outcomes and their impact on obesity-related comorbidities, using the corresponding 6-month follow-up period of the *Surg-First* group as a reference. Subsequently, the *Step-Treat* group was compared to the *Surg-First* group over a two-year period, applying the same criteria to evaluate weight loss and the influence on obesity-associated comorbidities.

### Conservative Treatment

The conservative obesity treatment program followed a multidisciplinary approach and lasted six months. During this period, patients attended six scheduled consultations with a registered dietitian to receive individualised nutritional counseling. They were required to maintain a detailed dietary log, which was reviewed and discussed during each of the six consultations. Nutritional guidance aimed to achieve a daily energy deficit of 500 kcal, based on each patient’s calculated total energy expenditure. In addition, patients were required to engage in physical activity at least twice per week for a minimum of one hour per session. These sessions had to consist of structured endurance or full-body training. Patients were responsible for recording their physical activity in a logbook, which had to be countersigned by a trainer to confirm participation. Additionally, all patients were assessed at least once by a mental health professional, with further sessions provided as needed based on individual requirements. At the beginning of the conservative treatment, after three months, and upon completion at six months, patients were evaluated by an interdisciplinary medical team consisting of endocrinologists and bariatric surgeons to monitor progress and discuss further treatment options.

Adequate weight loss during conservative therapy was defined according to the current German guidelines as a reduction of more than 20% of the initial body weight [23].

### Operative Procedures

#### Sleeve Gastrectomy

SG was performed laparoscopically using a five-port (Kii® Fios®, Applied Medical, Rancho Santa Margarita, CA, USA) technique. The freeing of the greater curvature started 4 cm proximal of the pylorus and was extended to left crus of the diaphragm using a LigaSure™ dissector (LigaSure ATLAS™, Medtronic GmbH, Meerbusch, Germany). For calibration of the gastric sleeve a 36 F gastric tube (Stomach Tube 36 Ch, P.J. Dahlhausen & Co. GmbH, Cologne, Germany) was placed and the stomach was divided parallel to the tube and along the greater curvature using a stapling device (Endo GIA™ Ultra Universal Stapler, 45 mm black/60 mm purple cartridges with Tri-Staple Technology, Medtronic GmbH, Meerbusch, Germany). Finally, the staple line was oversewn with a running suture (PDS™ 3–0, Ethicon GmbH, Norderstedt, Germany).

#### Roux-en-Y Gastric Bypass

For RYGB a laparoscopic 6-port technique (Kii® Fios®, Applied Medical, Rancho Santa Margarita, CA, USA) was used. After exposure of the left crus of the diaphragm the gastric pouch (about 30 ml) was created by dividing the stomach with a 45 mm purple cartridge horizontally and two 60 mm cartridges vertically (Endo GIA™ Ultra Universal Stapler, purple cartridges with Tri-Staple Technology, Medtronic GmbH, Meerbusch, Germany). A side-to-side jejuno-jejunostomy was established using one white 45 mm white cartridge (white cartridges with Tri-Staple Technology, Medtronic GmbH, Meerbusch, Germany), resulting in a 50–100 cm biliopancreatic and a 100–150 cm alimentary limb. The remaining defect was closed by a running suture (PDS™ 3–0, Ethicon GmbH, Norderstedt, Germany).

The gastro-jejunostomy was created using a transorally inserted anvil (DST Series™ EEA™ OrVil™, Medtronic GmbH, Meerbusch, Germany) with a 25 mm circular stapler (EEA™ Circular Stapler with DST Series™, Medtronic GmbH, Meerbusch, Germany). Subsequently the anastomosis was oversewn with simple interrupted stitches (PDS™ 3–0, Ethicon GmbH, Norderstedt, Germany).

### Definitions of Obesity-Related Co-Morbidities

#### Hypertension

Hypertension was defined as the use of antihypertensive medication before MBS. A decrease in the number of antihypertensive medications was rated as improvement and discontinuation of medication as remission. Worsening was defined as an increase in the number of antihypertensive medications.

#### Type 2 Diabetes Mellitus (T2DM)

T2DM was defined as the use of a diabetes medication or an or an HbA1_c_ level ≥ 6.5% in the absence of medication. According to the American Diabetes Association (ADA) consensus report, improvement was defined as a reduction in diabetes medication or discontinuation of medication with an HbA1_c_ remaining ≥ 6.5%. Remission was defined as the discontinuation of all diabetes medication with an HbA1_c_ < 6.5% sustained for at least three months without glucose-lowering medications [26]. An increase in the number of diabetes medications was considered as worsening.

#### Dyslipidemia

Medication for dyslipidemia or LDL-C > 115.8 mg/dl without medication was rated as dyslipidemia. Improvement was defined according to the European Society of Cardiology/European Atherosclerosis Society (ESC/EAS) guidelines by reduced medication or discontinued medication, but LDL-C > 115.8 mg/dl. Remission was defined by discontinued medication and LDL-C < 115.8 mg/dl. An initiation of medication or an increase in a preexisting medication was judged as worsening [27].

#### Obstructive Sleep Apnea Syndrome (OSAS)

The presence of OSAS was defined as the necessity for continuous positive airway pressure (CPAP) therapy. Discontinuation of CPAP therapy during follow-up was considered as improvement in OSAS.

### Quality of Life (QOL)

Assessment of the quality of life was carried out on the basis of the Moorehead-Ardelt Quality of Life Questionnaire II. The questionnaire is a standardised tool designed to assess the QOL in individuals who have undergone MBS. It evaluates six key domains: self-esteem, physical activity, social interactions, work performance, sexual functioning, and overall satisfaction with food-related experiences on scale form −3 to + 3 [28].

### Overall Outcome

To compare the overall outcome the SF-BARI QOL Score (Swiss-Finnish BARIatric Metabolic Outcome Score Quality of Life) was calculated. The SF-BARI QOL Score is a composite tool for standardized MBS outcome measurement. The Score includes %TWL, 4 obesity-related comorbidities (T2DM, hypertension, dyslipidemia, and obstructive sleep apnea) and complications (by Comprehensive Complication Index (CCI) and QOL (by Moorehead-Ardelt Quality of Life Questionnaire II) [28, 29]. Depending on the score outcomes are categorized as “Excellent” (≥ 150 pts), “Very Good” (125 to < 150 pts), “Good” (75 to < 125 pts), “Fair” (40 to < 75 pts) and “Suboptimal” (< 40 pts).

### Statistical Analysis

Statistical analysis was carried out using R (Version 4.3.1, R Foundation for Statistical Computing, Vienna, Austria). Data was tested for normal distribution with the Shapiro–Wilk test. For comparison of normally distributed data, a two-tailed unpaired t-test was used. Comparison of non-normally distributed data and ordinal-scaled data was performed by the Mann–Whitney-U test. In the case of repeated testing, the Holm correction method was applied to adjust for multiple comparisons.

For analysis of differences between nominal data, a χ^2^ test was used. If the number of events was smaller than five, a Fisher’s exact test was performed.

To compare obesity-associated comorbidities between treatment groups, Firth logistic regression was performed. Covariates included age, sex, and BMI at baseline. For diabetes, baseline HbA1c was included as an additional covariate; for dyslipidemia, baseline LDL-C was additionally considered.

A *p*-value < 0.05 was considered statistically significant.

## Results

### Study Population and Demographics

During the study period, a total of 918 patients presenting to our Bariatric Centre were index presentations. 149 patients (16.2%) were excluded from the study due to either loss to follow-up or a history of prior MBS. Among the remaining patients, 376 (48.9%) initiated conservative therapy, while 393 (51.1%) underwent primary bariatric surgery.

**Fig. 1:**
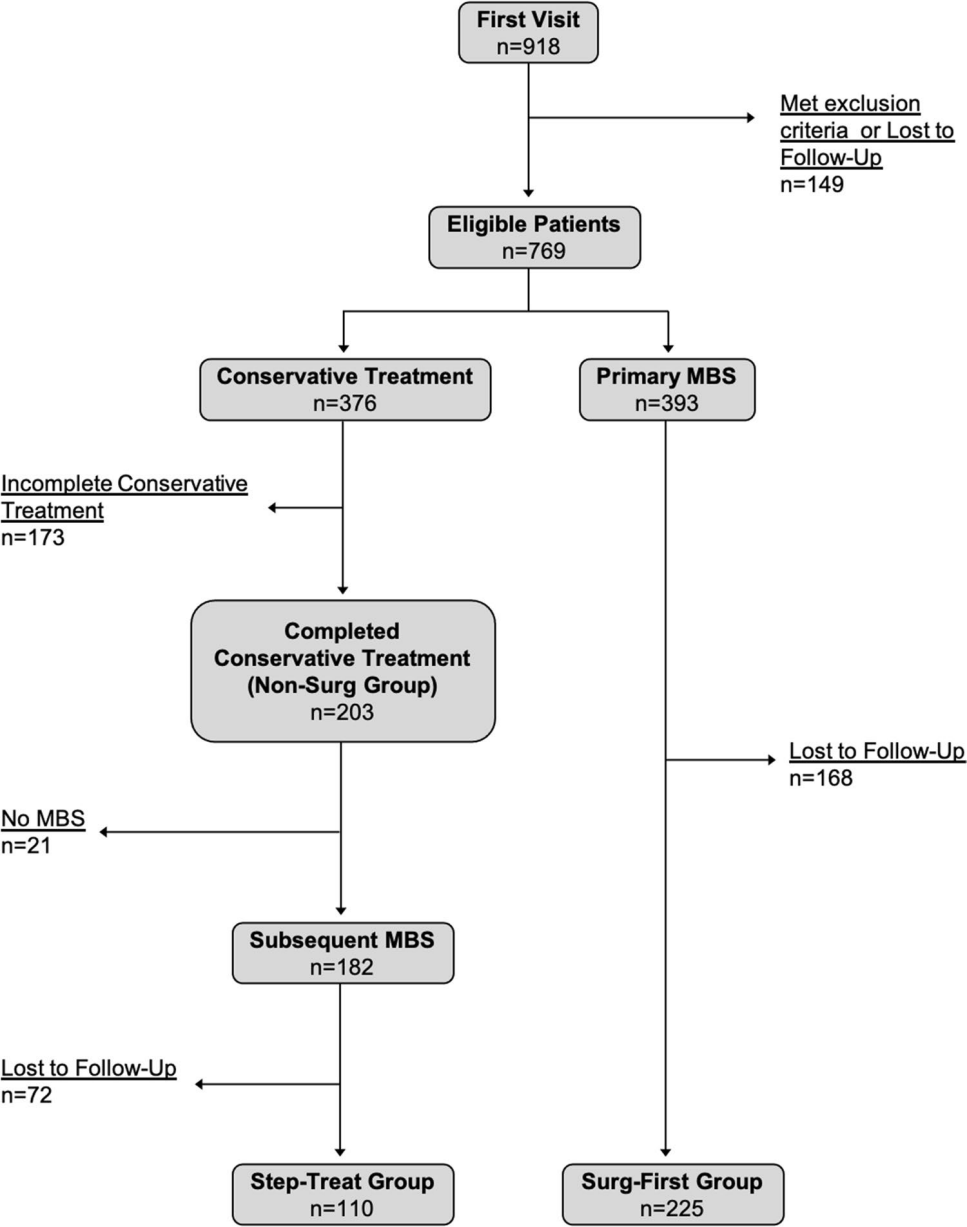
Patient flow in the study

Of the 376 patients who started conservative therapy, 203 (54.0%) completed the program and were included in the analysis (*Non-Surg* group). 173 patients (46.0%) discontinued treatment and were lost to follow-up. 182 out of 203 patients (89.7%) who completed conservative therapy subsequently proceeded to bariatric surgery. Of these, 110 patients (60.4%) had complete follow-up data and were included in the *Step-Treat* group. Among the patients who underwent primary MBS, 225 (57.3%) had complete follow-up data and were included in the *Surg-First* group (Fig. 1).

Age was similar across groups. The median age in the *Non-Surg* group was 40 years (IQR 30 to 49), 41 years (IQR 31.75 to 49) in the *Step-Treat* group, and 42 years (IQR 33 to 50) in the *Surg-First* group. Statistical comparisons revealed no significant differences in age between *Non-Surg* and *Surg-First* groups, nor between *Step-Treat* and *Surg-First* groups. Sex distribution showed a predominance of female patients in all groups. The proportion of females was 63.1% in the *Non-Surg* group (female-to-male ratio 1.7:1), 71.8% in the *Step-Treat* group (2.5:1), and 69.3% in the *Surg-First* group (2.3:1). There was no statistically significant difference in female representation between *Non-Surg* and *Surg-First*, nor between *Step-Treat* and *Surg-First* (Table 1).

**Table 1:** Baseline characteristics of the study groups

	Non-Surg	Step-Treat	Surg-First						
N	203	110	225	Non-Surg vs. Surg-First			Step-treat vs. Surg-First		
				*P* value	OR	95% CI	*P* value	OR	95% CI
Demographics									
Age (years)	40 (30 to 49)	41 (31,75 to 49)	42 (33 to 50)	0.09	N/A	N/A	0.40	N/A	N/A
Female (n, %)	128 (63.1)	79 (71.8)	156 (69.3)	0.18	0.75	0.50 to 1.12	0.70	1.13	0.68 to 1.85
Male (n, %)	75 (36.9)	31 (28.2)	69 (30.7)						
Ratio (f:m)	1.7:1	2.5:1	2.3:1	N/A	N/A	N/A	N/A	N/A	N/A
Weight Data									
Weight (kg)	164 (150 to 179)	160.5 (150 to 182.5)	162 (148 to 177)	0.69	N/A	N/A	0.55	N/A	N/A
BMI (kg/m^2^)	54.4 (51.7 to 59)	55.15 (54 to 56.3)	54.8 (51.4 to 60.2)	0.79	N/A	N/A	0.31	N/A	N/A
Comorbidities									
Hypertension (n, %)	80 (39.4)	57 (51.8)	128 (56.9)	< 0.001	0.49	0.33 to 0.72	0.41	0.81	0.51 to 1.29
T2DM (n, %)	33 (16.3)	23 (20.9)	61 (27.1)	0.007	0.52	0.33 to 0.84	0.23	0.72	0.41 to 1.22
Dyslipidemia (n, %)	82 (40.4)	47 (42.7)	84 (37.3)	0.55	1.14	0.77 to 1.69	0.34	1.25	0.79 to 2.01
OSAS (n, %)	38 (18.7)	25 (22.7)	45 (20)	0.81	0.92	0.56 to 1.49	0.47	1.22	0.70 to 2.09
ASA Classification									
II (n,%)	18 (8.9)	9 (8.2)	10 (4.4)	0.95	1.12	0.50 to 2.51	0,26	1.92	0.76 to 4.86
III (n,%)	181 (89.2)	100 (90.9)	194 (86.2)	0.44	1.31	0.73 to 2.35	0.29	0.63	0.29 to 1.33
IV (n,%)	4 (1.9)	1 (0.9)	21 (9.3)	0.002	0.19	0.07 to 0.58	0.0019	0.09	0.01 to 0.67
Metabolic Parameters									
HbA1_C_ (%)	6.8 (6.5 to 7.45)	7.1 (± 0.91)	7 (6.5 to 7.45)	0.31	N/A	N/A	0.64	N/A	N/A
LDL-C (mg/dl)	132 (121 to 145)	131 (121 to 147)	130 (120.3 to 154)	0.64	N/A	N/A	0.72	N/A	N/A

Normally distributed data are presented as mean ± standard deviation (SD), non-normally distributed data as median (IQR)

### Baseline Weight Data and Obesity-Related Comorbidities

Baseline weight and BMI were similar across treatment groups. Median weight was 164 kg (IQR, 150–179) in the *Non-Surg* group (*n* = 203), 160.5 kg (IQR, 150–182.5) in the *Step-Treat* group (*n* = 110), and 162 kg (IQR, 148–177) in the *Surg-First* group (*n* = 225). Median BMI was 54.4 (IQR, 51.7–59.0), 55.2 (IQR, 54.0–56.3), and 54.8 (IQR, 51.4–60.2), respectively. There were no statistically significant differences in weight or BMI between the *Non-Surg* and *Surg-First* groups or between the *Step-Treat* and *Surg-First* groups.

All patients were classified as ASA II to IV. ASA II was observed in 8.9% of patients in the Non-Surg group and in 4.4% of those in the Surg-First group. ASA III was present in 89.2% of Non-Surg patients and in 86.2% of Surg-First patients. ASA IV was identified in 1.9% of the Non-Surg group compared to 9.3% in the Surg-First group. No significant differences were found for ASA II or ASA III. However, ASA IV was significantly more frequent in the Surg-First group (OR 0.19, 95% CI 0.07–0.58, *P* = 0.002).

Hypertension was more prevalent in the *Surg-First* group (56.9%) than in the *Non-Surg* group (39.4%) (*P* < 0.001; OR, 0.49; 95% CI, 0.33–0.72). No significant difference was observed between the *Step-Treat* and *Surg-First*. The prevalence of type 2 diabetes was higher in the *Surg-First* group (27.1%) compared with the *Non-Surg* group (16.3%) (*P* = 0.007; OR, 0.52; 95% CI, 0.33–0.84). There was no significant difference between the *Step-Treat* and *Surg-First* groups. Median HbA1c was 6.8% (IQR, 6.5–7.45) in the *Non-Surg* group, 7.1% (SD, 0.91) in the *Step-Treat* group, and 7.0% (IQR, 6.5–7.45) in the *Surg-First* group with no statistical significant differences between the groups. Dyslipidemia was present in 40.4% of the *Non-Surg* group, 42.7% of the *Step-Treat* group, and 37.3% of the *Surg-First* group. Median LDL-C was 132 mg/dL (IQR, 121–145) in the *Non-Surg* group, 131 mg/dL (IQR, 121–147) in the *Step-Treat* group, and 130 mg/dL (IQR, 120.3–154) in the *Surg-First*. Differences in prevalence and LDL-C were not statistically significant. Obstructive sleep apnea syndrome was present in 18.7% of the *Non-Surg* group, 22.7% of the *Step-Treat* group, and 20.0% of the *Surg-First* group, with no significant differences between groups (Table 1).

### Conservative Treatment versus Primary MBS (Non-Surg vs. Surg-First Group)

#### Weight Outcomes and QOL

**Fig. 2:**
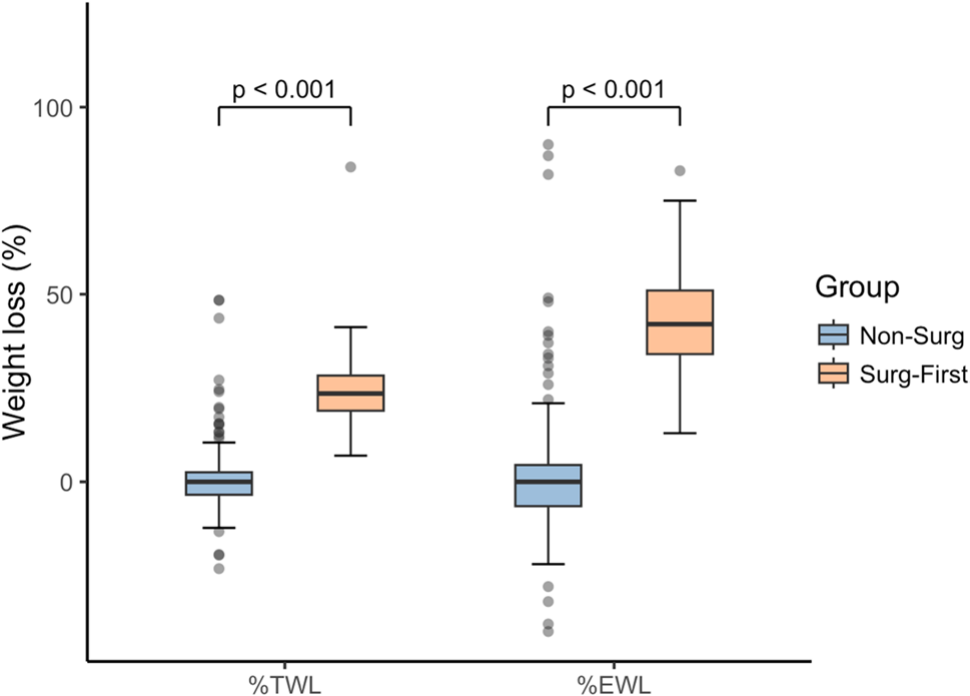
Percentage Total Weight Loss and Percentage Excess Weight Loss for the Non-Surg and Surg-First group 6 months after treatment. Boxplots display the median (center line), interquartile range (box), and values within 1.5 × IQR from the quartiles (whiskers); data points beyond this range are shown as outliers. Abbreviations: %TWL, Percentage Total Weight Loss; %EWL, Percentage Excess Weight Loss

Patients in the *Surg-First* group exhibited significantly greater weight loss compared to the *Non-Surg* group. Median weight loss was − 37 kg (IQR, − 47 to − 31) in the *Surg-First* group and 0 kg (IQR, − 4 to 6) in the *Non-Surg* group (*P* < 0.001). Correspondingly, median BMI was significantly lower in the *Surg-First* group at 42.6 kg/m^2^ (IQR, 38.3–46.6) versus 54.7 kg/m^2^ (IQR, 51.2–59.0) in the *Non-Surg* group (*P* < 0.001). The *Surg-First* group achieved a significantly higher median total weight loss (%TWL) of 23.6% (IQR, 19.0–28.4) compared to 0% (IQR, − 3.5 to 2.6) in the *Non-Surg* group (*P* < 0.001). Similarly, excess weight loss (%EWL) was 42% (IQR, 34–51) in the *Surg-First* group and 0% (IQR, − 7 to 5) in the *Non-Surg* group (*P* < 0.001). No statistically significant differences were observed in the baseline metabolic parameters HbA1c and LDL-C between groups (Table 2 and Fig. 2).

**Table 2:** Weight outcomes, changes in metabolic parameters and Quality of Life in the Non-Surg and Surg-First group 6 months after treatment

	Non-Surg	Surg-First	*P* value
n	203	225	
Weight Data			
Weight loss (kg)	0 (−4 to 6)	−37 (−47 to −31)	< 0.001
BMI (kg/m^2^)	54.7 (51.2 to 59)	42.6 (38.3 to 46.6)	< 0.001
%TWL (kg)	0 (−3.5 to 2.6)	23.6 (19 to 28.4)	< 0.001
%EWL (%)	0 (−7 to 5)	42 (34 to 51)	< 0.001
Metabolic Parameters			
HbA1_C_ (%)	6.7 (6.5 to 7.8)	6.6 (5.8 to 7.2)	0.15
LDL-C (mg/dl)	132 (121 to 150)	138.5 (121.8 to 157)	0.21
Quality of Life	0.6 (−1.4 to 0.3)	1.3 (0.3 to 2.1)	< 0.001

Normally distributed data are presented as mean ± standard deviation (SD), non-normally distributed data as median (IQR)

Among patients in the *Non-Surg* group, 36 (17.7%) showed no change in body weight during conservative treatment, and 92 (45.4%) experienced weight gain. A total of 69 patients (34.0%) achieved weight reduction; however, only 6 patients (3.0%) reached an adequate degree of weight loss.

Median quality-of-life scores were significantly higher in the *Surg-First* group (1.3, IQR 0.3 to 2.1) compared with the *Non-Surg* group (0.6, IQR −1.4 to 0.3, P < 0.001) (Table 2).

#### Changes in Obesity-Related Comorbidities

**Fig. 3:**
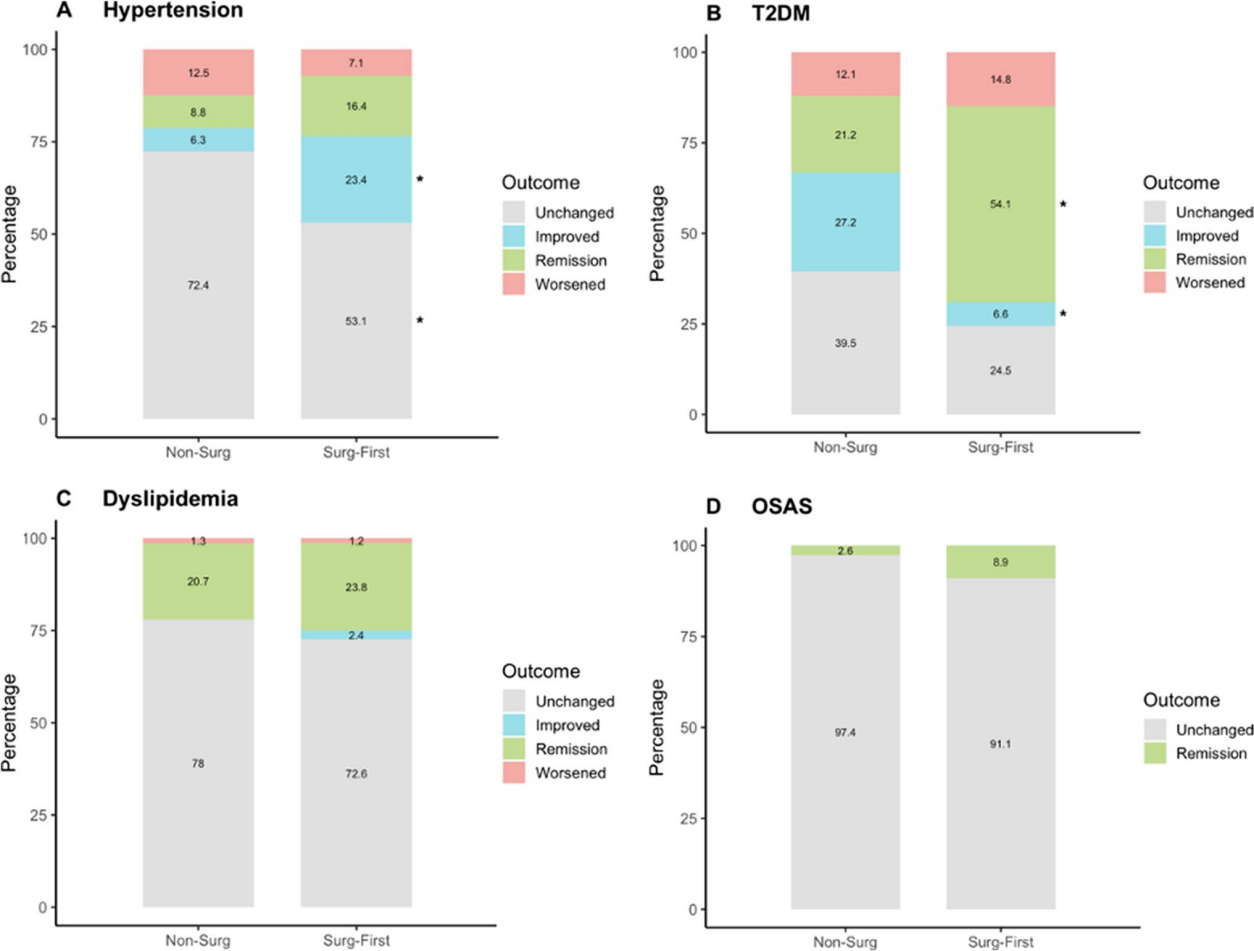
Outcomes in obesity-related comorbidities in the Non-Surg and Surg-First group 6 months after treatment. Values represent the percentage of patients in each respective outcome category; * *p* < 0.05 vs Non-Surg. Abbreviations: T2DM, Type 2 Diabetes; OSAS, Obstructive Sleep Apnea Syndrome

Hypertension improved significantly in the *Surg-First* group (17.1% difference; OR 4.00, 95% CI 1.59–11.98; *P* = 0.002), whereas no significant improvement in hypertension was observed in the *Non-Surg* group (19.3% difference; OR 0.46, 95% CI 0.25–0.84; *P* = 0.011). T2DM remission was substantially more frequent in the *Surg-First* group (32.9% difference; OR 12.17, 95% CI 3.65–52.16; *P* < 0.001). Comparing patients with persisting T2DM the patients in the *Non-Surg* group showed a more significant improvement than in the *Surg-First* group (20.6% difference; OR 0.06, 95% CI 0.01–0.30; *P* < 0.001). De novo T2DM occurred exclusively in the Non-Surg group (6.5% difference; OR 11.28, 95% CI 1.44–88.37; *P* < 0.001). No significant differences were found between groups for dyslipidemia. Most cases remained unchanged, and de novo cases did not differ significantly. For OSAS, a modest difference in remission favored the *Surg-First* group but did not reach statistical significance. The vast majority of patients in both groups showed unchanged OSAS (Table 3 and Fig. 3).

**Table 3:** Outcomes of obesity-related comorbidities in the Non-Surg and Surg-First group 6 months after treatment

	Non-Surg (n, %)	Surg-First (n, %)	Absolute difference, % (95% CI)	OR (95% CI)	*P* Value
Hypertension	89 (43.8)	113 (50.2)	6.4 (−16.3 to 3.5)	0.77 (0.52 to 1.15)	0.21
Remission	7 (8.8)	21 (16.4)	7.6 (−1.3 to 16.6)	2.27 (0.96 to 5.95)	0.06
Improved	5 (6.3)	30 (23.4)	17.1 (8.1 to 26.2)	4.00 (1.59 to 11.98)	0.002
Unchanged	58 (72.4)	68 (53.1)	−19.3 (−32.4 to −6.3)	0.46 (0.25 to 0.84)	0.011
Worsened	10 (12.5)	9 (7.1)	−5.5 (−14.0 to 3.0)	0.48 (0.18 to 1.24)	0.13
De Novo	16 (13)	6 (6.2)	−6.8 (−14.5 to 0.8)	0.54 (0.19 to 1.41)	0.21
T2DM	37 (18.2)	28 (12.9)	−5.3 (−2.1 to 12.7)	1.5 (0.86 to 2.66)	0.14
Remission	7 (21.2)	33 (54.1)	32.9 (14.2 to 51.6)	12.17 (3.65 to 52.16)	< 0.001
Improved	9 (27.2)	4 (6.6)	−20.6 (−37.1 to −4.3)	0.06 (0.01 to 0.30)	< 0.001
Unchanged	13 (39.5)	15 (24.5)	−15 (−34.7 to 5.1)	0.39 (0.14 to 1.03)	0.06
Worsened	4 (12.1)	9 (14.8)	2.7 (−11.6 to 16.9)	1.09 (0.34 to 3.96)	0.89
De Novo	11 (6.5)	0	−6.5 (−9.8 to −2.7)	11.28 (1.44–88.37)	< 0.001
Dyslipidemia	85 (41.9)	94 (41.8)	−0.1 (−9.4 to 9.6]	0.98 (0.67 to 1.5)	> 0.99
Remission	17 (20.7)	20 (23.8)	3.1 (−9.6 to 15.7)	1.30 (0.63 to 2.73)	0.48
Improved	0	2 (2.4)	2.4 (−0.9 to 5.6)	5.89 (0.38 to 883.62)	0.22
Unchanged	64 (78)	61 (72.6)	−5.4 (−18.5 to 7.7)	0.69 (0.34 to 1.41)	0.31
Worsened	1 (1.3)	1 (1.2)	−0.1 (−3.3 to 3.3)	0.92 (0.07 to 11.29)	0.94
De Novo	20 (16.5)	30 (21.3)	4.8 (−4.3 to 12.5)	1.24 (0.66 to 2.33)	0.51
OSAS	43 (21.2)	46 (20.4)	0.8 (−7.4 to 8.9)	1.05 (0.64 to 1.71)	0.91
Remission	1 (2.6)	4 (8.9)	−6.3 (−16.0 to 3.5)	0.32 (0.03 to 1.87)	0.22
Unchanged	37 (97.4)	41 (91.1)	6.3 (−3.5 to 16.0)	3.13 (0.53 to 33.16)	0.22
De Novo	6 (3.6)	5 (2.8)	−0.8 (−4.6 to 2.8)	0.77 (0.22 to 2.61)	0.68

*T2DM* Type 2 Diabetes, *OSAS* Obstructive Sleep Apnea Syndrome

Metabolic Parameters were comparable between groups. Median HbA1c was 6.7% (IQR, 6.5–7.8) in the *Non-Surg* group and 6.6% (IQR, 5.8–7.2) in the *Surg-First* group (*P* = 0.15). Similarly, LDL-C levels did not differ significantly, with 132 mg/dL (IQR, 121–150) in the *Non-Surg* group versus 138.5 mg/dL (IQR, 121.8–157) in the *Surg-First* group (*P* = 0.21) (Table 2).

### Conservative Treatment Followed by MBS versus Primary MBS (*Step-Treat* vs. *Surg-First* Group)

#### Operative Details and Postoperative Outcomes

Median operating time was 78.5 min (IQR, 64.8–94.5) in the *Step-Treat* group and 79 min (IQR, 66.5–94.5) in the *Surg-First* group, with no statistically significant difference between groups. Median length of hospital stay was 6 days (IQR, 5–7) in both groups, also without a significant difference. Length of ICU stay was significantly longer in the Step-Treat group, with a median of 0.8 days (SD 2.6) compared with 0.4 days (SD 0.6) in the Surg-First group (*P* = 0.001).

Follow-up data was available for 142 of 215 patients (66.0%) in the *Step-Treat* group and for 155 of 221 patients (70.1%) in the *Surg-First* group. There was no statistically significant difference in follow-up completion between the two groups (OR 1.14, 95% CI 0.80–1.63; *P* = 0.52).

SG was the most common procedure, performed in 96 of 110 patients (87.2%) in the *Step-Treat* group and in 189 of 225 patients (84.0%) in the *Surg-First* group. RYGB was performed in 14 patients (12.8%) and 36 patients (16.0%), respectively. The distribution of procedure type did not differ significantly between groups. Previous abdominal surgery was more frequently reported in the Surg-First group (46.7%) than in the Step-Treat group (33.6%), representing a statistically significant difference (OR 0.58, 95% CI 0.36–0.93; *P* = 0.03). There was no difference in the proportion of previous open (48.6% vs 51.4%) or laparoscopic (51.4% vs 48.6%) procedures between groups.

All patients were ASA II-IV. ASA II was observed in 8.2% versus 4.4%, ASA III in 90.9% versus 86.2%, and ASA IV in 0.9% versus 9.3% of patients in the *Step-Treat* and *Surg-First* groups, respectively. There was no significant difference regarding ASA II or ASA III, while ASA IV was significantly more common in the Surg-First group (OR 0.09, 95% CI 0.01–0.65, *P* = 0.0019). Anticoagulation therapy and platelet inhibitor use showed no significant differences between groups.

Overall, postoperative complications occurred in 36 of 110 patients (32.6%) in the *Step-Treat* group and in 59 of 225 (26.2%) in the *Surg-First* group. This difference was not statistically significant. Complications were classified according to the Clavien-Dindo grading system. Grade I complications were observed in 4 *Step-Treat* patients (3.6%) vs 23 *Surg-First* patients (10.2%), Grade II in 15 (13.6%) vs 26 (11.5%), and Grade IIIa in 3 (2.7%) vs 2 (0.9%). These differences were not statistically significant. Grade IIIb complications occurred more frequently in the *Step-Treat* patients (13 of 110 [11.8%]) than in the *Surg-First* patients (8 of 225 [3.6%]), representing a statistically significant difference (*P* < 0.001). One Grade IVb complication (0.9%) occurred in the *Step-Treat* group and none in the *Surg-First* group. No Grade V complications were reported in either group (Table 4). Rates of specific postoperative complications were largely comparable between groups. However, stenosis occurred significantly more frequently in the Step-Treat group (*P* = 0.03), while no other complication differed significantly (Table 5). Among patients with major complications (Clavien-Dindo grade ≥ IIIb), the most frequent causes in the Step-Treat group were stenosis (*n* = 6), bleeding (*n* = 3), and small bowel perforation (*n* = 2). In addition, there was one case each of leak, incarcerated incisional hernia, and small bowel infarction secondary to portal vein thrombosis. In the Surg-First group, major complications included conversion to RYGB for therapy-refractory reflux (*n* = 3) and stenosis (*n* = 2), as well as one case each of leak, bleeding, and deep wound infection.

**Table 4:** Operative and postoperative metrics of Step-Treat und Surg-First group

	Step-Treat	Surg-First	*P* value	OR	95% CI
n	110	225			
Operating time (min)	78.5 (64.75 to 94.5)	79 (66.5 to 94.5)	0.39	N/A	N/A
Length of stay (d)	6 (5 to 7)	6 (5 to 7)	0.25	N/A	N/A
Length of ICU stay (d)	0.8 (2.6)	0.4 (0.6)	0.001	N/A	N/A
Procedure (n, %)					
SG	96 (87.2)	189 (84)	0.43	0.77	0.39 to 1.5
RYGB	14 (12.8)	36 (16)			
Previous abdominal surgery (n, %)	37 (33.6)	105 (46.7)	0.03	0.58	0.36 to 0.93
open	18 (48.6)	54 (51.4)	0,92	0,89	0.42 to 1.89
laparoscopic	19 (51.4)	51 (48.6)			
Anticoagulation therapy	5 (4.5)	11 (4.9)	0.3	2.03	0.68 to 6.03
Platelet inhibitor use	6 (5.5)	9 (4.0)	0.58	0,72	0.25 to 2.08
Complications (n, %)	36 (32.6)	59 (26.2)	0.27	1.37	0.83 to 2.25
I	4 (3.6)	23 (10.2)	0.06	0.33	0.11 to 0.98
II	15 (13.6)	26 (11.5)	0.71	1.21	0.61 to 2.39
IIIa	3 (2.7)	2 (0.9)	0.41	3.13	0.51 to 18.9
IIIb	13 (11.8)	8 (3.6)	0.007	3.64	1.46 to 9.06
IVa	0 (0)	0 (0)	N/A	N/A	N/A
IVb	1 (0.9)	0 (0)	0.33	0.83	0.97 to 1.0
V	0 (0)	0 (0)	N/A	N/A	N/A
SF Bari QOL Score	97.9 ± 34.4	109.9 ± 36.3	0.002	N/A	N/A
Excellent (n, %)	3 (2.7)	5 (2.2)	> 0.99	1.23	0.29 to 5.26
Very good (n, %)	7 (6.4)	44 (19.6)	0.003	0.28	0.12 to 0.64
Good (n, %)	58 (52.7)	125 (55.6)	0.71	0.89	0.56 to 1.41
Fair (n, %)	39 (35.5)	45 (20)	0.004	2.2	1.32 to 3.66
Suboptimal (n, %)	3 (2.7)	6 (2.6)	0.63	0.56	0.14 to 2.28

Normally distributed data are presented as mean ± standard deviation (SD), non-normally distributed data as median (IQR)

The complications are reported according to the Clavien-Dindo classification. The percentage of each category refers to the total number of complications

**Table 5:** Postoperative complications in the Step-Treat und Surg-First group

	Step-Treat	Surg-First	*P* value	OR	95% CI
n	110	225			
Complication (n, %)					
Bleeding	4 (3.6)	2 (0.9)	0.09	4.21	0.76 to 23.3
Leakage	1 (0.9)	1 (0.5)	0.55	2.06	0.13 to 33.2
Wound infection	2 (1.8)	3 (1.3)	0.67	1.37	0.23 to 8.32
Stenosis	6 (5.5)	2 (0.9)	0.03	0.33	0.11 to 0.97
Marginal ulcer	0	2 (0.9)	> 0.99	0.4	0.01 to 8.5
GERD	10 (9.1)	17 (7.6)	0.67	1.22	0.54 to 2.77
Dumping syndrome	3 (2.7)	7 (3.1)	> 0.99	0.87	0.22 to 3.44
Incisional hernia	2 (1.8)	5 (2.2)	> 0.99	0.81	0.16 to 4.27
Other	8 (7.2)	20 (8.9)	0.68	0.8	0.34 to 1.89

*GERD* Gastroesophageal reflux disease

Mean SF-BARI QOL score was significantly lower in *the Step-Treat* Group than in the *Surg-First* group (97.9 ± 34.4 vs. 109.9 ± 36.3, *P* = 0.002). Based on the SF-BARI QOL score an excellent outcome was reported in 3 of 110 patients (2.7%) in the Step-Treat group and in 5 of 225 (2.2%) in the Surg-First group; the difference was not statistically significant. Very good outcomes were reported more frequently in the Surg-First group (44 patients [19.6%]) than in the Step-Treat group (7 patients [6.4%]), which was statistically significant (*P* = 0.003). A good outcome was documented in 58 patients (52.7%) vs 125 (55.6%), without a significant difference. A fair outcome occurred more often in the Step-Treat group (39 patients [35.5%]) compared with the Surg-First group (45 patients [20%]), a statistically significant difference (*P* = 0.004). Suboptimal outcomes were rare and comparable between groups (3 [2.7%] vs 6 [2.6%]), with no significant difference (Table 4).

#### Weight Outcomes and QOL

**Fig. 4:**
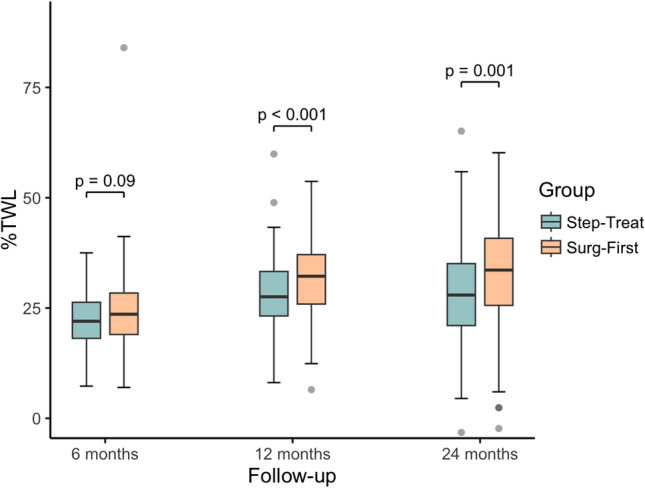
Percentage Total Weight Loss for the Step-Treat and Surg-First group 6, 12 and 24 months after treatment. Boxplots display the median (center line), interquartile range (box), and values within 1.5 × IQR from the quartiles (whiskers); data points beyond this range are shown as outliers. Abbreviations: %TWL, Percentage Total Weight Loss

**Fig. 5:**
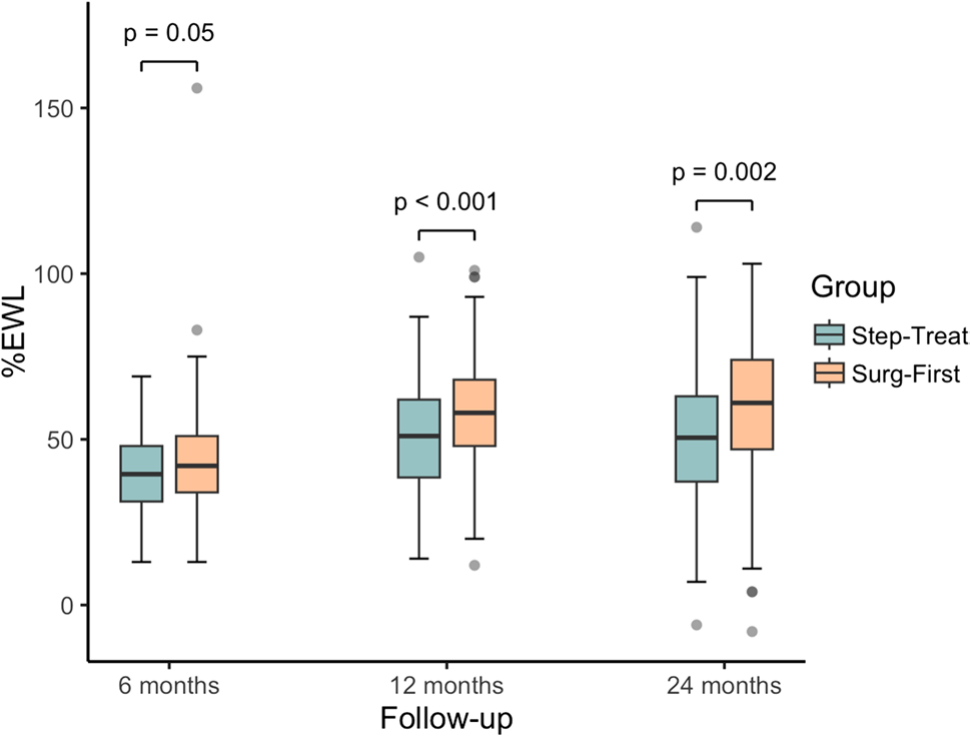
Percentage Excess Weight Loss for the Step-Treat and Surg-First group 6, 12 and 24 months after treatment. Boxplots display the median (center line), interquartile range (box), and values within 1.5 × IQR from the quartiles (whiskers); data points beyond this range are shown as outliers. Abbreviations: %EWL, Percentage Excess Weight Loss

After 6 months of treatment, weight loss, BMI, %TWL, and %EWL were not significantly different between groups. Mean weight loss was 36.8 ± 10.6 kg in the *Step-Treat* group and median 37 kg (IQR, 31–47) in the *Surg-First* Group (*P* = 0.18). BMI values were comparable (43.0 [IQR, 39.6–48.7] vs. 42.6 [IQR 38.3–46.5]; *P* = 0.09), as were %TWL (22.1 ± 5.1% vs. 23.6% [IQR, 19.0–28.4]; *P* = 0.09). %EWL was not significant (40.2 ± 12.0% vs. 42.0% [34–51]; *P* = 0.05). At 12 months, all weight-related outcomes were significantly improved in the *Surg-First* group. Median weight loss was 52 kg (IQR, 40.5–63) compared to 45 kg (IQR, 39–54) in the *Step-Treat* group (*P* = 0.001). BMI was significantly lower (37.6 [IQR, 33.9–42.4] vs. 40.9 ± 7.1; *P* = 0.003), as were %TWL (31.8 ± 8.3% vs. 28.2 ± 8.2%; *P* < 0.001) and %EWL (58.3 ± 15.8% vs. 51.2 ± 16.1%; *P* < 0.001). At 24 months, the *Surg-First* group continued to show superior results: weight loss was significantly greater (54.4 ± 29.3 kg vs. 46 kg [IQR, 33.5–55.5]; *P* = 0.001), BMI was significantly lower (36.5 [IQR, 32.4–42.1] vs. 40.8 ± 8.3; *P* = 0.002), and %TWL (32.7 ± 10.9% vs. 28.3 ± 11.0%; *P* = 0.001) as well as %EWL (59.6 ± 20.1% vs. 51.6 ± 20.7%; *P* = 0.002) remained significantly higher (Table 6, Figs. 4, and 5).

No significant differences in QOL were observed between groups at 6, 12 and 24 months (Table 6).

**Table 6:** Weight outcomes, changes in metabolic parameters and Quality of Life in the Step-Treat and Surg-First group 6, 12 and 24 months after MBS

	Step-Treat	Surg-First	*P* value
n	110	225	
6 Months			
Weight Data			
Weight loss (kg)	36.8 ± 10.6	37 (31 to 47)	0.18
BMI (kg/m^2^)	43 (39.6 to 48.7)	42.6 (38.3 to 46.5)	0.09
%TWL (kg)	22.1 ± 5.1	23.6 (19 to 28.4)	0.09
%EWL (%)	40.2 ± 12	42 (34 to 51)	0.05
Metabolic Parameters			
HbA1_C_ (%)	5.9 ± 0.6	6.6 (5.8 to 7.2)	0.1
LDL-C (mg/dl)	136.7 ± 22.6	138.5 (121.8 to 157)	0.67
Quality of Life	1.3 (0.8 to 1.9)	1.3 (0.3 to 2.1)	0.41
12 Months			
Weight Data			
Weight loss (kg)	45 (39 to 54)	52 (40.5 to 63)	0.001
BMI (kg/m^2^)	40.9 ± 7.1	37.6 (33.9 to 42.4)	0.003
%TWL (kg)	28.2 ± 8.2	31.8 ± 8.3	< 0.001
%EWL (%)	51.2 ± 16.1	58.3 ± 15.8	< 0.001
Metabolic Parameters			
HbA1_C_ (%)	6 (5.2 to 6.2)	6.4 (6.1 to 6.9)	0.02
LDL-C (mg/dl)	129 (121 to 143)	133 (120 to 151)	0.87
Quality of Life	1.2 (0.4 to 2)	1.4 (0.4 to 2.2)	0.23
24 Months			
Weight Data			
Weight loss (kg)	46 (33.5 to 55.5)	54.4 ± 29.3	0.001
BMI (kg/m^2^)	40.8 ± 8.3	36.5 (32.4 to 42.1)	0.002
%TWL (kg)	28.3 ± 11	32.7 ± 10.9	0.001
%EWL (%)	51.6 ± 20.7	59.6 ± 20.1	0.002
Metabolic Parameters			
HbA1_C_ (%)	6.2 ± 0.7	6.7 (6 to 7)	0.34
LDL-C (mg/dl)	131.5 (122.8 to 145)	128 (116.3 to 143.8)	0.1
Quality of Life	1.3 (0.3 to 2)	1.5 (0.3 to 2.2)	0.34

Normally distributed data are presented as mean ± standard deviation (SD), non-normally distributed data as median (IQR)

#### Changes in Obesity-Related Comorbidities

No significant group differences were observed at 6 months for any of the analyzed comorbidities. By 12 months, remission of OSAS was significantly more frequent in the *Step-Treat* group (7.1% difference; OR 26.44, 95% CI 2.51–35.90; *P* = 0.004). Stable dyslipidemia was also significantly more frequent in the *Step-Treat* group at this time point (14.2% difference; OR 0.43, 95% CI 0.17–1.00; *P* = 0.049). All other comparisons at 12 months showed no significant differences. At 24 months, the disparity in unchanged dyslipidemia further increased, remaining significantly more frequent in the *Step-Treat* group compared to *Surg-First* (22.3% difference; OR 0.39, 95% CI 0.18–0.81; *P* = 0.012). No further significant differences in OSAS outcomes were observed at this time point. All remaining outcomes at 24 months were not statistically different.

**Fig. 6:**
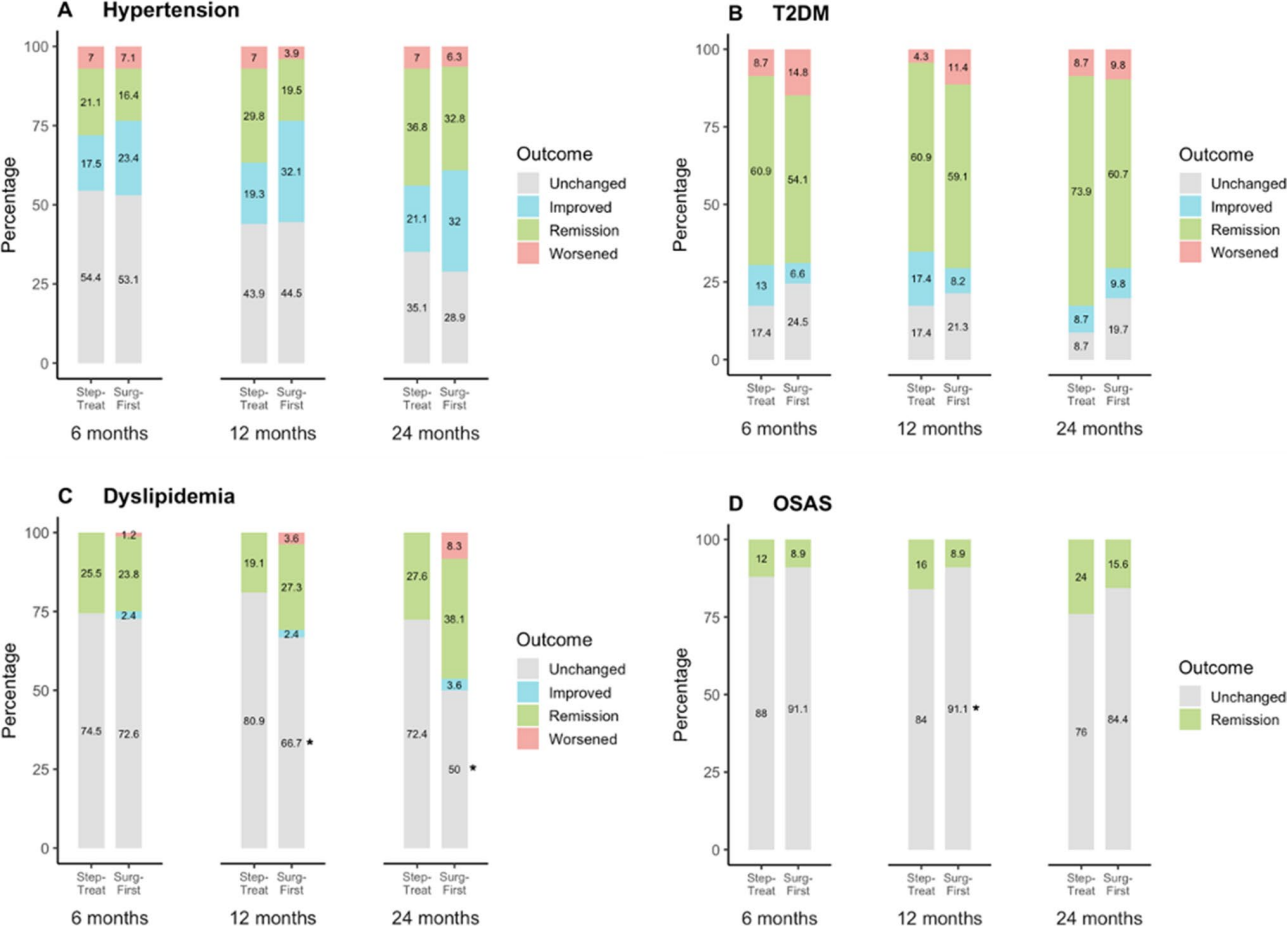
Outcomes in obesity-related comorbidities in the Step-Treat and Surg-First group 6, 12 and 24 months after treatment. Values represent the percentage of patients in each respective outcome category; * *p* < 0.05 vs Step-Treat. Abbreviations: T2DM, Type 2 Diabetes; OSAS, Obstructive Sleep Apnea Syndrome

Regarding metabolic parameters, no significant difference in HbA1c was observed between groups at 6 months (median 5.7% [IQR, 5.4–6.1] vs. 5.7% [5.3–6.3]; *P* = 0.64). However, at 12 months, HbA1c was significantly lower in the *Surg-First* group (5.6% [IQR, 5.3–5.9]) compared to the *Step-Treat* group (6.0% [IQR, 5.5–6.6]; *P* < 0.001), and this difference persisted at 24 months (5.5% [IQR, 5.2–5.9] vs. 5.9% [5.3–6.4]; *P* = 0.001) (Table 7 and Fig. 6).

For LDL-C, levels were comparable at 6 months (median 2.4 mmol/L [IQR, 2.1–2.9] in the *Step-Treat* group vs. 2.3 mmol/L [IQR, 1.8–2.7] in the *Surg-First* group; *P* = 0.08). However, at 12 months and 24 months, LDL-C was significantly lower in the *Surg-First* group (2.2 mmol/L [IQR, 1.8–2.6] vs. 2.4 [2.1–2.9]; *P* = 0.009 at 12 M, and 2.1 [1.7–2.5] vs. 2.5 [2.1–3.0]; *P* < 0.001 at 24 M) ( Table 6).

## Discussion

This study demonstrates that, among patients with a BMI ≥ 50 kg/m^2^, conservative therapy results in significantly inferior weight loss compared to surgical intervention. These findings align with a broad body of evidence demonstrating the superior efficacy of bariatric surgery in achieving sustained weight loss in individuals with severe obesity [5, 17]. In contrast, bariatric surgery is associated with substantial and durable weight reduction, as well as improved survival and comorbidity remission [8, 9].

In the present cohort, only 3% of patients in the non-surgical group achieved clinically relevant weight loss through conservative treatment. Furthermore, 46% of patients dropped out of the conservative therapy program, which likely reflects frustration with the minimal short-term results typically associated with non-surgical measures. This high dropout rate is a clinically meaningful signal that suggests a discrepancy between policy expectations and patient needs. These observations raise concerns that mandatory conservative therapy prior to surgery may delay effective treatment, potentially worsening medical and economic outcomes by allowing further disease progression (Table 7).

**Table 7:** Outcomes of obesity-related comorbidities in the Step-Treat and Surg-First group 6, 12 and 24 months after MBS

	Step-Treat (n, %)	Surg-First (n, %)	Absolute difference, % (95% CI)	OR (95% CI)	*P* Value
6 Months					
Hypertension	49 (44.5)	113 (50.2)	5.7 (−17.7 to 6.4)	0.8 (0.49 to 1.29)	0.353
Remission	12 (21.1)	21 (16.4)	−4.7 (−17.0 to 7.7)	0.75 (0.35 to 1.69)	0.482
Improved	10 (17.5)	30 (23.4)	5.9 (−6.4 to 18.2)	1.37 (0.64 to 3.16)	0.427
Unchanged	31 (54.4)	68 (53.1)	−1.3 (−16.8 to 14.3)	0.98 (0.52 to 1.82)	0.943
Worsened	4 (7)	9 (7.1)	−0.1 (−8.0 to 8.0)	0.90 (0.29 to 3.21)	0.867
De Novo	4 (7.5)	6 (6.2)	−1.3 (−7.5 to 7.6)	0.87 (0.25 to 3.30)	0.823
T2DM	9 (8.2)	29 (12.9)	4.7 (−12.1 to 2.7)	0.6 (0.24 to 1.37)	0.271
Remission	14 (60.9)	33 (54.1)	−6.8 (−30.3 to 16.8)	0.89 (0.29 to 2.68)	0.834
Improved	3 (13)	4 (6.6)	−6.4 (−21.6 to 8.6)	0.25 (0.03 to 1.53)	0.132
Unchanged	4 (17.4)	15 (24.5)	7.1 (−11.7 to 26.1)	1.26 (0.37 to 4.96)	0.717
Worsened	2 (8.7)	9 (14.8)	6.1 (−8.5 to 20.6)	1.72 (0.41 to 10.01)	0.477
De Novo	0	0	N/A	N/A	N/A
Dyslipidemia	47 (42.7)	94 (41.8)	−0.9 (−11 to 12.9)	1.04 (0.64 to 1.69)	0.906
Remission	12 (25.5)	20 (23.8)	−1.7 (−17.2 to 13.7)	0.97 (0.43 to 2.25)	0.941
Improved	0	2 (2.4)	2.4 (−0.9 to 5.6)	2.84 (0.18 to 395.83)	0.494
Unchanged	35 (74.5)	61 (72.6)	−1.9 (−17.5 to 13.8)	0.87 (0.38 to 1.94)	0.738
Worsened	0	1 (1.2)	1.2 (−1.1 to 3.5)	1.69 (0.09 to 257.89)	0.743
De Novo	12 (19)	30 (21.3)	2.3 (−7.6 to 12.9)	1.28 (0.62 to 2.75)	0.512
OSAS	24 (21.8)	46 (20.4)	−1.4 (−8.7 to 11.4)	1.09 (0.59 to 1.95)	0.776
Remission	3 (12)	4 (8.9)	−3.1 (−18.3 to 12.1)	0.70 (0.16 to 3.38)	0.640
Unchanged	22 (88)	41 (91.1)	3.1 (−12.1 to 18.3)	1.43 (0.30 to 6.41)	0.640
De Novo	2 (2.4)	5 (2.8)	0.4 (−3.5 to 4.3)	0.95 (0.22 to 5.43)	0.951
12 Months					
Hypertension	43 (39.1)	107 (47.6)	8.5 (−20.4 to 3.4)	0.71 (0.43 to 1.15)	0.161
Remission	17 (29.8)	25 (19.5)	−10.3 (−24.0 to 3.4)	0.59 (0.29 to 1.23)	0.156
Improved	11 (19.3)	41 (32.1)	12.8 (−0.3 to 25.8)	1.90 (0.92 to 4.19)	0.085
Unchanged	25 (43.9)	57 (44.5)	0.6 (−14.8 to 16.2)	1.02 (0.55 to 1.92)	0.946
Worsened	4 (7)	5 (3.9)	−2.1 (−10.5 to 4.3)	0.53 (0.14 to 2.03)	0.337
De Novo	3 (5.7)	4 (4.1)	−1.6 (−6.7 to 4.7)	0.68 (0.16 to 3.15)	0.606
T2DM	9 (8.2)	25 (11.1)	2.9 (−10.2 to 4.3)	0.71 (0.28 to 1.65)	0.448
Remission	14 (60.9)	36 (59.1)	−1.8 (−25.3 to 21.6)	1.24 (0.39 to 3.86)	0.712
Improved	4 (17.4)	5 (8.2)	−9.2 (−26.1 to 7.8)	0.32 (0.07 to 1.45	0.137
Unchanged	4 (17.4)	13 (21.3)	3.9 (−14.7 to 22.5)	0.92 (0.26 to 3.57)	0.892
Worsened	1 (4.3)	7 (11.4)	7.1 (−4.4 to 18.7)	2.18 (0.39 to 22.64)	0.396
De Novo	0	0	N/A	N/A	N/A
Dyslipidemia	51 (46.4)	90 (40)	−6.4 (−5.6 to 18.4)	1.3 (0.8 to 2.11)	0.29
Remission	9 (19.1)	23 (27.3)	8.2 (−6.5 to 23.0)	1.79 (0.75 to 4.57)	0.191
Improved	0	2 (2.4)	2.4 (−0.9 to 5.6)	2.54 (0.18 to 347.32)	0.509
Unchanged	38 (80.9)	56 (66.7)	−14.2 (−29.3 to 0.9)	0.43 (0.17 to 1.00)	0.049
Worsened	0	3 (3.6)	3.6 (−0.4 to 7.5)	2.82 (0.22 to 393.57)	0.464
De Novo	13 (25.4)	29 (20.6)	−4.8 (−11.0 to 10.3)	0.97 (0.48 to 2.05)	0.736
OSAS	23 (20.9)	46 (20.4)	−0.5 (−9.3 to 10.2)	1.03 (0.56 to 1.86)	> 0.99
Remission	4 (16)	4 (8.9)	−7.1 (−23.7 to 9.5)	26.44 (2.51 to 35.90)	0.004
Unchanged	21 (84)	41 (91.1)	7.1 (−9.5 to 23.7)	0.04 (0.00 to 0.40)	0.004
De Novo	2 (2.4)	5 (2.8)	0.4 (−3.4 to 4.3)	0.96 (0.22 to 5.48)	0.961
24 Months					
Hypertension	39 (35.5)	89 (39.6)	4.9 (−15.8 to 7.6)	0.84 (0.51 to 1.38)	0.55
Remission	21 (36.8)	42 (32.8)	−4.0 (−19.0 to 10.9)	0.89 (0.44 to 1.79)	0.732
Improved	12 (21.1)	41 (32)	10.9 (−2.3 to 24.3)	1.71 (0.82 to 3.72)	0.152
Unchanged	20 (35.1)	37 (28.9)	−6.2 (−20.9 to 8.5)	0.74 (0.38 to 1.44)	0.369
Worsened	4 (7)	8 (6.3)	−0.7 (−8.6 to 7.1)	0.83 (0.26 to 3.00)	0.763
De Novo	3 (5.7)	3 (3.1)	−2.6 (−7.0 to 3.2)	0.51 (0.10 to 2.47)	0.386
T2DM	6 (5.5)	24 (10.7)	5.2 (11.7 to 1.3)	0.48 (0.16 to 1.27)	0.154
Remission	17 (73.9)	37 (60.7)	−13.2 (−35.0 to 8.5)	0.70 (0.21 to 2.16)	0.535
Improved	2 (8.7)	6 (9.8)	1.1 (−12.6 to 14.9)	0.75 (0.15 to 4.54)	0.729
Unchanged	2 (8.7)	12 (19.7)	11.0 (−4.3 to 26.2)	1.71 (0.42 to 9.68)	0.475
Worsened	2 (8.7)	6 (9.8)	1.1 (−12.6 to 14.9)	1.05 (0.22 to 6.44)	0.952
De Novo	0	0	N/A	N/A	N/A
Dyslipidemia	36 (32.7)	80 (35.6)	2.9 (−14.3 to 8.6)	0.88 (0.53 to 1.47)	0.627
Remission	13 (27.7)	32 (38.1)	10.4 (−6.0 to 26.9)	1.70 (0.80 to 3.77)	0.170
Improved	0	3 (3.6)	3.6 (−0.4 to 7.5)	3.59 (0.33 to 483.84)	0.333
Unchanged	34 (72.3)	42 (50)	−22.3 (−39.0 to −5.7)	0.39 (0.18 to 0.81)	0.012
Worsened	0	7 (8.3)	8.3 (2.4 to 14.2)	7.54 (0.86 to 990.45)	0.073
De Novo	12 (19)	28 (19.9)	0.9 (−9.5 to 10.3)	1.05 (0.52 to 2.25)	0.893
OSAS	22 (20)	43 (19.1)	−0.9 (−8.9 to 10.6)	1.06 (0.57 to 1.94)	0.883
Remission	6 (24)	7 (15.6)	−8.4 (−28.3 to 11.4)	0.59 (0.18 to 1.99)	0.384
Unchanged	19 (76)	38 (84.4)	8.4 (−11.4 to 28.3)	1.70 (0.50 to 5.68)	0.384
De Novo	3 (3.5)	5 (2.8)	−0.7 (−5.0 to 3.7)	0.68 (0.17 to 3.02)	0.588

*T2DM* Type 2 Diabetes, *OSAS* Obstructive Sleep Apnea Syndrome

Beyond weight loss, surgically treated patients demonstrated significantly better outcomes in terms of obesity-related comorbidities. Notably, the Surgery-First group exhibited significantly higher rates of remission and improvement for both hypertension and T2DM including a markedly lower rate of de novo T2DM. These findings are supported by previous trials showing metabolic benefits of bariatric surgery. In particular, randomized-controlled trials by Schauer et al. have repeatedly demonstrated superior T2DM control and remission after surgery compared to medical therapy alone [30, 31]. Such benefits underscore the role of early surgical intervention in mitigating long-term metabolic risk. Mechanistically, bariatric procedures—particularly those involving gastrointestinal bypass—promote favorable changes in gut hormones, insulin sensitivity, and beta-cell function, which likely contribute to these observed benefits.

Importantly, our data show that a stepwise approach, where surgery follows conservative therapy (*Step-Treat group*), was not associated with improved outcomes in obesity-related comorbidities or QOL compared to immediate surgery (*Surg-First* group). On the contrary, weight loss in this group was significantly lower at 12 and 24 months than in the Surgery-First group. This difference may have downstream clinical consequences, especially in comorbidities not analyzed here. For example, sustained weight loss has been shown to slow progression of osteoarthritis and coronary artery disease [32–35]. As a result, the inferior weight loss in the Stepwise-Treatment group may have broad negative implications. The clinical relevance of these differences was further supported by analysis of overall outcomes. According to the SF-BARI QOL score, the proportion of patients achieving “very good” or “excellent” results was significantly higher in the *Surg-First* group. Moreover, total SF-BARI QOL score was significantly lower in the *Step-Treat* group, further supporting the advantage of immediate surgery.

Interestingly, major postoperative complications (Clavien-Dindo ≥ IIIb) were more frequent in the Step-Treat group. This higher complication rate may, at least in part, explain the longer ICU stay observed in this group. The difference occurred despite comparable demographics and a similar incidence of obesity-associated comorbidities between groups, as well as no significant differences in the proportion of patients receiving oral anticoagulation or platelet inhibitor therapy. Notably, the Surg-First group even had a significantly higher proportion of patients classified as ASA IV, indicating greater baseline surgical risk. In addition, a greater proportion of patients in the Surg-First group had undergone previous abdominal surgery, which is generally considered a risk factor for postoperative complications. One possible explanation is that the delay in surgical treatment allowed for further disease progression, increasing technical complexity and surgical risk. Another contributing factor may be metabolic instability induced by failed conservative treatment, leading to impaired wound healing or increased vulnerability to postoperative complications. It is also conceivable that prolonged exposure to high inflammatory burden, psychological stress from delayed surgery, or cumulative nutritional deficiencies during conservative treatment contributed to worsened postoperative outcomes. Furthermore, factors such as the extent of intra-abdominal adhesions or the size of the left hepatic lobe, both potentially relevant to surgical complexity and complication risk, could not be assessed due to the retrospective design of the study. However, the exact reasons for the higher complication rate in the Step-Treat group cannot be conclusively determined based on the available data. Other unmeasured factors may have influenced the results and warrant further investigation.

Several limitations must be acknowledged. The retrospective design entails selection bias, particularly regarding group allocation, which was influenced by patient preference and insurance policies. Another major limitation is that this study did not allow for analysis of long-term complications of bariatric surgery, particularly beyond 2 years postoperatively. Specifically, late complications such as marginal ulcers, gastroesophageal reflux disease, stenosis, and dumping syndrome, which are well-described sequelae of bariatric procedures, may be underestimated [36–38]. Likewise, no conclusions can be drawn regarding recurrent weight gain or recurrence of comorbidities that initially resolved, such as T2DM or hypertension, in the long-term follow-up. Additionally, most surgical patients in this cohort underwent sleeve gastrectomy, due to an institutional policy favoring this procedure in patients with BMI ≥ 50 kg/m^2^ based on technical feasibility and safety profiles [39, 40]. However, this may limit generalizability to centers that perform a higher proportion of Roux-en-Y gastric bypass (RYGB).

A further concern in this study and in bariatric surgery research more broadly, is the low follow-up rate. Although follow-up completion did not differ significantly between the *Step-Treat* and *Surg-First* groups, overall follow-up was suboptimal. This phenomenon is not unique to our dataset. Several studies have identified follow-up attrition as a major barrier to long-term outcome assessment in bariatric cohorts [13, 14, 41]. To mitigate this, various interventions have been proposed, including mobile applications, telehealth platforms, automatic reminder systems, and patient incentives [42–44]. Integration of such tools into clinical practice may help increase patient engagement and support data completeness in future studies.

In conclusion, our data support the notion that conservative therapy, either alone or as a prerequisite to surgery, is not beneficial in patients with BMI ≥ 50 kg/m^2^. Immediate surgical intervention results in superior outcomes regarding weight loss, comorbidity remission, and complication rates. These findings underscore the importance of reconsidering mandatory preoperative conservative therapy in this patient population. When feasible and medically appropriate, direct access to bariatric surgery should be prioritized for individuals with severe obesity to maximize health benefits.

To further guide clinical decision-making randomized controlled trials comparing immediate surgery with stepwise treatment strategies, as well as long-term outcomes are needed. Moreover, standardized conservative treatment protocols are needed to determine whether optimized non-surgical interventions could effectively prepare selected patients for surgery. Finally, the potential role of emerging pharmacologic therapies, particularly GLP-1 receptor agonists, warrants investigation with regard to their impact on conservative obesity treatment strategies and surgical decision-making.

## Conclusion

In patients with BMI ≥ 50 kg/m^2^, immediate bariatric surgery leads to significantly better outcomes in terms of weight loss, comorbidity remission, and complication rates compared to conservative or stepwise treatment. These findings support early surgical intervention as the preferred approach in this population.

## Data Availability

The data that support the findings of this study are not publicly available due to the sensitive nature of patient health information. Data are stored securely at the University Medical Center Hamburg-Eppendorf and are available from the corresponding author upon reasonable request and with appropriate ethical and institutional approvals.
